# Aerobic-Strength Exercise Improves Metabolism and Clinical State in Parkinson’s Disease Patients

**DOI:** 10.3389/fneur.2017.00698

**Published:** 2017-12-22

**Authors:** Patrik Krumpolec, Silvia Vallova, Lucia Slobodova, Veronika Tirpakova, Matej Vajda, Martin Schon, Radka Klepochova, Zuzana Janakova, Igor Straka, Stanislav Sutovsky, Peter Turcani, Jan Cvecka, Ladislav Valkovic, Chia-Liang Tsai, Martin Krssak, Peter Valkovic, Milan Sedliak, Barbara Ukropcova, Jozef Ukropec

**Affiliations:** ^1^Institute of Experimental Endocrinology, Biomedical Research Center, Slovak Academy of Sciences, Bratislava, Slovakia; ^2^Institute of Pathological Physiology, Faculty of Medicine, Comenius University, Bratislava, Slovakia; ^3^Institute of Sports Medicine and Physical Education, Faculty of Medicine, Slovak Medical University in Bratislava, Bratislava, Slovakia; ^4^Faculty of Physical Education and Sports, Comenius University, Bratislava, Slovakia; ^5^High Field MR Centre, Department of Biomedical Imaging and Imaged-Guided Therapy, Medical University of Vienna, Vienna, Austria; ^6^Christian Doppler Laboratory for Clinical Molecular Imaging, MOLIMA, Medical University of Vienna, Vienna, Austria; ^7^2nd Neurology Department, Faculty of Medicine, Comenius University & University Hospital Bratislava, Bratislava, Slovakia; ^8^1st Neurology Department, Faculty of Medicine, Comenius University & University Hospital Bratislava, Bratislava, Slovakia; ^9^Oxford Centre for Clinical Magnetic Resonance Research (OCMR), BHF Centre of Research Excellence, University of Oxford, Oxford, United Kingdom; ^10^National Cheng-Kung University, Tainan, Taiwan; ^11^Division of Endocrinology and Metabolism, Department of Internal Medicine III, Medical University of Vienna, Vienna, Austria

**Keywords:** exercise training, Parkinson’s disease, energy metabolism, ^31^P-MRS, muscle metabolism

## Abstract

Regular exercise ameliorates motor symptoms in Parkinson’s disease (PD). Here, we aimed to provide evidence that exercise brings additional benefits to the whole-body metabolism and skeletal muscle molecular and functional characteristics, which might help to explain exercise-induced improvements in the clinical state. 3-months supervised endurance/strength training was performed in early/mid-stage PD patients and age/gender-matched individuals (*n* = 11/11). The effects of exercise on resting energy expenditure (REE), glucose metabolism, adiposity, and muscle energy metabolism (^31^P-MRS) were evaluated and compared to non-exercising PD patients. Two muscle biopsies were taken to determine intervention-induced changes in fiber type, mitochondrial content, and expression of genes related to muscle energy metabolism, as well as proliferative and regenerative capacity. Exercise improved the clinical disability score (MDS-UPDRS), bradykinesia, balance, walking speed, REE, and glucose metabolism and increased muscle expression of energy sensors (AMPK). However, the exercise-induced increase in muscle mass/strength, mitochondrial content, type II fiber size, and postexercise phosphocreatine (PCr) recovery (^31^P-MRS) were found only in controls. Nevertheless, MDS-UPDRS was associated with muscle AMPK and mechano-growth factor (MGF) expression. Improvements in fasting glycemia were positively associated with muscle function and the expression of *Sirt1* and *Cox7a1*, and the parameters of fitness/strength were positively associated with the expression of *MyHC2, MyHC7*, and *MGF*. Moreover, reduced bradykinesia was associated with better muscle metabolism (maximal oxidative capacity and postexercise PCr recovery; ^31^P-MRS). Exercise training improved the clinical state in early/mid-stage Parkinson’s disease patients, including motor functions and whole-body metabolism. Although the adaptive response to exercise in PD was different from that of controls, exercise-induced improvements in the PD clinical state were associated with specific adaptive changes in muscle functional, metabolic, and molecular characteristics.

**Clinical Trial Registration:**

www.ClinicalTrials.gov, identifier NCT02253732.

## Key Points

- Aerobic-strength exercise training improved the clinical state in early/mid-stage Parkinson’s disease patients, specifically motor functions and bradykinesia.
- Training improved the whole-body glucose and energy metabolism in PD patients and induced changes in muscle metabolic, functional, and molecular characteristics in both PD patients and controls.
- The adaptive response to exercise in Parkinson’s disease patients was distinctly different from that observed in healthy controls, while no changes were found in control non-exercising PD patients.
- Exercise-induced effects on muscle metabolic state and fiber type were associated with bradykinesia, glucose tolerance, resting energy expenditure (REE), and with improvements in the PD clinical state.
- REE, free-living ambulatory activity, and muscle strength explained 72.5% of the variability in bradykinesia.

## Introduction

Parkinson’s disease (PD) is a chronic neurodegenerative disorder that affects ~1% of the population >60 years of age (1). The clinical profile includes a variety of motor and non-motor symptoms, with increased risk of falls and a dramatic impact on the quality of life and functional independence (2, 3). The progressive nature of the disease and generally symptomatic treatment require new strategies in early stage disease management (4). Mounting evidence shows that physical activity has unequivocal benefits for PD patients (5–7), with a potential to lower the disability score (MDS-UPDRS) (8). Regular exercise has the potential to improve underlying metabolic derangements, including inflammation, mitochondrial dysfunction, and glucose metabolism. A higher incidence of glucose intolerance and type 2 diabetes (>50%) was found among PD patients (9–11) and the presence of glucose intolerance has been shown to accelerate the progression of PD (12), with a sedentary lifestyle considered one of the common denominators of neurodegeneration and metabolic dysfunction (13). Skeletal muscle is the organ of motion and the largest organ in our body, corresponding to 35–40% of the whole-body mass. Muscle is a major player in the whole-body energy metabolism, with the ability to communicate with other cells, tissues, and organs to maintain functional integrity and energy homeostasis. Improving muscle functional state in PD by regular exercise could, therefore, improve the whole-body functional capacity, slowing down disease progression. It is known that the dopaminergic deficit, central to the pathophysiology of PD, leads to increased tonic inhibition of the thalamus, thus reducing the excitatory drive for the motor cortex (14). This, in turn, may affect the cortical activation of muscles (15, 16), which may be manifested as muscle weakness. Such a reduction in the force generated during muscle contraction is indicative of strength and/or movement speed. There is a proposed relationship between muscle weakness and bradykinesia (17, 18), one of the primary motor symptoms of PD (19).

In this work, muscle ^31^P-MRS, as well as measures of balance and muscle power, were used to evaluate the exercise-related changes in muscle metabolic and functional state. When combined with the assessment of muscle fiber type, mitochondrial content, and the expression of key metabolic genes, they provided a comprehensive view of the exercise-induced adaptive changes that are associated with improvements of whole-body metabolism and motor disability in Parkinson’s disease patients.

In our study, we evaluated effects of 3-month supervised aerobic-strength training intervention on the whole-body and muscle metabolism, clinical disabilities, physical fitness and muscle functional, morphological and molecular characteristics in patients with PD, and age/gender/BMI-matched controls.

## Materials and Methods

The study population consisted of 13 sedentary seniors and 12 sedentary patients with Parkinson’s disease (duration: 7.1 ± 3.9 years, Hoehn–Yahr 1–3). All PD patients received standard care from a neurologist and were on appropriate PD medication (l-DOPA/carbidopa, dopamine agonists, MAO inhibitors). All volunteers underwent medical examination, including blood tests, complex metabolic phenotyping, aerobic physical fitness and muscle strength assessments, nutritional profiling, free-living physical activity assessment, and motoric/balance testing before/after training intervention. The capacity to undergo intervention was assessed/approved by a cardiologist, and patients with known uncontrolled or late-stage cardiac, renal, liver, oncologic, or other chronic diseases were excluded. Parkinson’s disease patients and age/gender/BMI-matched controls (*n* = 11/11) completed the 3-month-supervised aerobic-strength exercise intervention. The study population was complemented by Parkinson’s disease patients who did not undergo training intervention (*n* = 5, disease duration 7.8 ± 4.8 years, Hoehn–Yahr 2–3, age 62.4 ± 9.8 years, M/F 4/1). The clinical study flow chart is shown in (Figure S1 in Supplementary Material).

The protocol was approved by the Ethics Committee of the University Hospital Bratislava and conformed to the ethical guidelines of the Helsinki declaration of 1964 (2000 revision). All individuals signed a written, informed consent prior to the study. The small patient sample is a major limitation of this study, which was attributable to the complex nature of the study protocol.

### Combined Strength/Endurance Supervised Exercise Training

A 3-month combined strength/endurance supervised exercise training program was designed and performed at the Faculty of Physical Education and Sports, Comenius University in Bratislava. 1-h training sessions, preceded by a 10 min warm-up and followed by cool-down/stretching exercises, were performed three times/week: one session of aerobic dancing, and two sessions of brisk walking/Nordic walking/stationary bicycling (60–70% VO_2_max, individualized according to the Rockport test), combined with resistance training of major muscle groups, based on muscle functional testing, starting at 50–60% of one repetition maximum (1RM) training was performed under the supervision of experienced exercise physiologists and progressive load increase paralleled improvements in performance (~2% 1RM/week). Adherence to the training program was monitored and regularly encouraged, resulting attendance was >85%.

### Metabolic Phenotyping

BMI and waist circumference were recorded. Body composition was assessed by bioelectric impedance (Omron-BF511, Japan) between 08:00 a.m. and 9:00 a.m., after an overnight fast and void. Volume and distribution (subcutaneous/visceral) of abdominal fat was determined using five consecutive MRI slices (9-cm wide abdominal region) centered between L4/L5 (20) (3T-Trio, Siemens, Germany) and evaluated with the IDL ver.6.3 (Exelis VIS-Inc., USA) and ImageJ 1.48e (NIH, USA). A 2-h oral glucose tolerance test (oGTT) was performed in the morning, after an overnight fast and 30 min after intravenous cannula insertion (Surflo-W, Belgium), to determine glucose tolerance and calculate the insulin resistance index (HOMA-IR). Blood samples were drawn before, 30, 60, 90, and 120 min after the ingestion of 75 g glucose and were used to determine levels of circulating glucose, insulin, total and high-density lipoprotein (HDL) cholesterol, triglycerides, and hsCRP (Alpha-Medical, Slovakia) using commercially available methods. The atherogenic index was calculated with the formula (total_cholesterol-HDL_cholesterol)/HDL_cholesterol. REE and metabolic substrate preference (RQ) (Ergostik, Geratherm-Respiratory, Germany) were assessed by indirect calorimetry in the fasted state.

### Physical Fitness, Muscle Strength, and Free-Living Ambulatory Activity

Cardiovascular/aerobic fitness was evaluated with the Rockport 1-mile walking test. After a short warm-up, subjects walked as briskly as possible for 1 mile (1,609 m) on a 400-m track. Heart rate (Polar RS300X, Finland) and time of completion (Witty, MicroGate, Italy) were electronically recorded and VO_2_max was calculated according to Ref. (21). Maximal isometric force and the rate of force development (RFD) were determined on a linear legpress in a semi-squat position (22); and maximum isometric torque of knee extensors and flexors was assessed with a knee dynamometer (S2P-Ltd., Ljubljana, Slovenia). Free-living ambulatory activity was monitored with accelerometers (Omron-Healthcare Co., Japan) for a minimum of three consecutive days with at least 12 h active-time recording. A standardized acute bicycling exercise was performed before and after training, using stationary bicycles and heart rate monitoring (Polar RS300X, 40 min at 70% HRmax).

### Unified Parkinson’s Disease Rating Scale and Motor Function Testing

The severity of Parkinson’s disease was evaluated by the Movement Disorder Society-Unified Parkinson’s Disease Rating Scale (MDS-UPDRS). All patients underwent examination in the “ON” state, after taking appropriate medication, with the difference between the “ON” and the “OFF” state being >30% (MDS-UPDRS). Specific subscores reflecting posture and gait, rigidity, tremor, and bradykinesia were calculated (Table S1 in Supplementary Material). The Berg Balance Scale (BBS) was used to assess a relevant change in balance (23) and the risk of falls.

### Skeletal Muscle Biopsy

Samples of the *m.vastus lateralis* were obtained by Bergström needle biopsy under local anesthesia in the fasted state, before/after the 3-month exercise program, as previously described (20). Muscle samples were immediately cleaned and frozen/stored in liquid nitrogen. A small, well-defined part of the muscle was embedded in TissueTek, frozen in 3-methylbuthane chilled by liquid nitrogen, and stored at −80°C for immunohistochemistry.

### Determination of Muscle Fiber Type in Native Tissue Sections

Transversal 6 µm cryosections were prepared. A myofibrillar ATPase activity assay was performed following preincubation with an acid (pH~4) solution that predominantly inhibited the myosin ATPase activity in fast glycolytic/fast oxidative (type 2B/2X/2A) fibers. The method is described in Ref. (24). Fiber type-specific fiber size and relative quantity were evaluated.

### Muscle Metabolism by ^31^P-MRS

^31^P-MRS was performed on a 7T scanner, using a dual-tuned ^1^H/^31^P surface coil (10 cm diameter, Rapid-Biomedical, Germany). Baseline intramyocellular concentrations of phosphorous metabolites were assessed at rest. The exercise challenge described previously (25) consisted of 6-min plantar flexion (Trispect, Ergospect, Austria), calibrated by individual maximal voluntary contraction (MVC). The muscle-group-specific (*m. gastrocnemius*) measurement of phosphocreatine (PCr) resynthesis during the 6-min recovery period yielded a time constant of PCr recovery (τ_PCr_) and maximal oxidative capacity (Q_max_) (26).

### RNA/DNA Isolation and qPCR

Total RNA/DNA was isolated from skeletal muscle using TriReagent (Molecular Research Center, Inc., USA). Purified (RNeasy Mini Kit, Qiagen, USA), DNAse-treated (Qiagen, USA) RNA was used for gene expression studies. Relative mtDNA content was determined as a ratio between markers of mitochondrial (ND1) and nuclear (RPL13a) genome. A High Flex RNA to cDNA kit was used (Qiagen, USA). Gene expression was measured by qRT-PCR (ABI7900HT, Applied Biosystems, USA), using either pre-designed TaqMan gene expression assays or a set of primers designed with PrimerExpress (Applied Biosystems, USA; Table S2 in Supplementary Material). Ribosomal protein L13a and 18S rRNA were used as internal reference genes to calculate dCt expression values.

### Statistical Analysis

Statistical analyses were performed using SAS Jump Statistics Software (USA) and G*Power software ver. 3.1.9.2. Data were tested for normal distribution. Paired *t*-test was used to assess the difference between baseline and postexercise variables. Unpaired *t*-test and general linear model were used to assess the differences between the intervention effects (delta follow-up baseline). More than two group differences were evaluated by ANOVA with the Tukey *post hoc* test. Results are given as means ± SD (unless indicated otherwise). Pearson correlation and a stepwise regression model were used to determine the association state between variables. Statistical significance was considered at *p* < 0.05, and for trends, *p* < 0.09.

## Results

### Characteristics of the Study Population

Eleven sedentary PD patients (MDS-UPDRS score 42.0 ± 6.5) were compared to age/gender/BMI-matched healthy controls. Key phenotypic characteristics are summarized in Table 1. A small group of PD patients who did not undergo exercise training intervention had a higher MDS-UPDRS disability score (52.0 ± 11.9, *p* = 0.045). However, obesity and metabolic phenotypes [BMI, body fat, subcutaneous, and visceral adiposity (MRI), muscle mass, HOMA-IR, 2-h glucose, circulating lipids, metabolic substrate preference (RQ), and REE] of this non-exercising control group were comparable to the pre-intervention state of PD patients who underwent exercise intervention. No physical fitness, muscle functional parameters, or muscle biopsies were obtained from non-exercising PD controls.

**Table 1:** Characteristics of the study population, aerobic fitness, and muscle functional, metabolic, and biochemical parameters.

	Seniors (pre-training)	Seniors (post-training)	PD (pre-training)	PD (post-training)
		
Age (years)	66.3 ± 2.2		62.9 ± 6.6	
Sex (M/F)	5/6		5/6	
**Body composition, blood pressure, and whole-body metabolism**				
Weight (kg)	75.3 ± 13.5	74.2 ± 13.1	79.3 ± 16.4	78.2 ± 17.0
BMI (kg m^−2^)	27.0 ± 3.9	26.6 ± 3.5	28.5 ± 5.1	28.1 ± 5.2
Body fat (%)	32.9 ± 12.4	29.9 ± 11.6	34.5 ± 10.3	33.5 ± 10.5
Subcutaneous adipose tissue (dm^3^)	3.01 ± 1.51	2.23 ± 0.88	3.15 ± 1.69	2.82 ± 1.19
Visceral adipose tissue (dm^3^)	1.93 ± 0.52	2.08 ± 8.89	2.38 ± 0.94	2.41 ± 0.88
Muscle mass (%)	28.3 ± 6.5	30.3 ± 6.0	28.3 ± 5.3	28.7 ± 5.4
Systolic BP, 24 h monitoring (mmHg)	135 ± 9	130 ± 13	123 ± 11	122 ± 13
Diastolic BP, 24 h monitoring (mmHg)	78 ± 7	78 ± 10	78 ± 9	76 ± 7
Heart rate, 24 h monitoring (min^−1^)	66 ± 5	64 ± 11	74 ± 6*	76 ± 5**
Fasting glucose (mmol l^−1^)	4.81 ± 0.37	4.79 ± 0.28	4.91 ± 0.35	**4.58 ± 0.28^#^**
2 h glucose (mmol l^−1^)	7.12 ± 1.86	7.18 ± 2.30	7.14 ± 1.84	5.03 ± 1.34^#,^*
Fasting Insulin (mIU l^−1^)	6.62 ± 3.54	6.24 ± 2.67	13.64 ± 11.08	10.04 ± 6.84
HOMA-IR	1.45 ± 0.89	1.33 ± 0.70	2.99 ± 2.30^♪^*	2.02 ± 1.48
hsCRP (mg l^−1^)	1.69 ± 1.01	1.40 ± 0.33	1.47 ± 1.13	1.41 ± 1.12
Total cholesterol (mmol l^−1^)	5.10 ± 0.89	5.11 ± 0.76	5.38 ± 1.24	5.16 ± 1.13
high-density lipoprotein-cholesterol (mmol l^−1^)	1.64 ± 0.54	1.75 ± 0.56	1.62 ± 0.37	1.80 ± 0.35
TAG (mmol l^−1^)	0.91 ± 0.36	0.95 ± 0.09	1.13 ± 0.46	1.02 ± 0.47
REE/24 hour (kcal.24 h^−1^)	1,188 ± 308	1,303 ± 251	1,116 ± 355	**1,461 ± 290^#^**
RQ (VCO_2_/VO_2_)	0.83 ± 0.03	0.84 ± 0.07	0.86 ± 0.09	0.82 ± 0.08
**Physical fitness and balance**				
VO_2_max (mlO_2_/kgBW/min)	32.7 ± 6.9	37.0 ± 9.6	30.4 ± 10.3	33.8 ± 9.8
Heart rate–rockport test (min^−1^)	141 ± 19	145 ± 14	131 ± 19	133 ± 12^♪^*
Walking speed–rockport test (m s^−1^)	1.8 ± 0.2	2.0 ± 0.3	1.7 ± 0.2	1.8 ± 0.3^♪^*
Steps per hour (during the active time)^b^	674 ± 81	732 ± 112	568 ± 70	480 ± 62^♪^*
Chair stand up test (s)	9.8 ± 1.7	**8.4 ± 1.2^#^**	10.3 ± 2.1	**8.8 ± 0.7^♪#^**
10 m pref. speed (s)	7.96 ± 0.85	**6.58 ± 0.58^###^**	7.98 ± 1.06	**6.97 ± 0.71^#^**
Berg Balance Scale score	55.3 ± 1.5	55.3 ± 1.6	50.4 ± 3.6**	**53.2 ± 2.3^#^**
**Muscle strength**				
Max voluntary knee flexion (N m^−1^)	135.9 ± 45.6	161.2 ± 49.1	130.2 ± 49.6	133.1 ± 56.0
Max voluntary knee extension (N m^−1^)	290.5 ± 99.3	298.1 ± 119.5	270.5 ± 117.7	289.4 ± 133.9
Max voluntary contract. legpress (N m^−1^)	1,027 ± 282	1,086 ± 254	706 ± 205*	750 ± 222**
Rate of force development^a^ (N ms^−1^)	23.3 ± 14.1	**28.7 ± 18.0^♪#^**	20.8 ± 12.7	21.9 ± 13.2^♪^*
**Biochemical parameters**				
Myoglobin, *baseline resting state* (μg l^−1^)	65.9 ± 22.2	63.0 ± 18.3	46.6 ± 12.8*	59.1 ± 7.0
Creatine kinase–*resting state* (μcat l^−1^)	2.14 ± 1.34	2.10 ± 1.31	1.39 ± 1.14	2.05 ± 1.24
Lactate–*baseline resting state* (mmol l^−1^)	1.68 ± 0.30	**0.68 ± 0.22^###^**	1.96 ± 0.37	**0.83 ± 0.24^###^**
Lactate–*acutely postexercise* (mmol l^−1^)	5.40 ± 1.92	3.38 ± 2.16	4.95 ± 1.75	**3.21 ± 1.79^#^**
Lactate–*60* *min postexercise* (mmol l^−1^)	2.56 ± 0.68	1.73 ± 1.03	2.22 ± 0.45	**1.08 ± 0.39^###^**
**Muscle metabolism and gene expression**				
τ_PCr_ (s)	44.5 ± 14.3	**30.8 ± 5.6^♪#^**	59.6 ± 25.4	52.3 ± 26.3^♪^*
Q_max_ (mM s^−1^)	0.57 ± 0.06	0.62 ± 0.1	0.47 ± 0.22	0.59 ± 0.12
PRKAA1 (dCt) 1,000×	1.0 ± 0.5	**1.9 ± 0.5^#^**	1.6 ± 0.5	**2.2 ± 0.4^♪#^**
FoxJ3 (dCt) 1,000×	6.0 ± 2.9	5.2 ± 1.4	6.3 ± 1.9	7.8 ± 1.1*
MyHC 2/MyHC7	0.95 ± 0,36	**1.44 ± 0.53^♪#^**	1.90 ± 0.41**	1.82 ± 0.14
MGF (dCt) 1,000×	0.6 ± 0.3	0.8 ± 0.04	0.4 ± 0.2	0.5 ± 0.1***
ND1/Rpl13a	1.00 ± 0.28	**1.53 ± 0.66^#^**	1.24 ± 0.55	1.26 ± 0.77

Data are expressed as average ± SEM, bold is used to highlight statistical significance; effect of exercise intervention is marked with ^#^p < 0.05, ^##^p < 0.01, ^###^p < 0.001; differences between PD patients and controls are marked with *p < 0.05, **p < 0.01, ***p < 0.001; ^♪^denotes trend p < 0.09.

*BP, blood pressure; REE, resting energy expenditure; RQ, respiratory quotient (metabolic substrate preference)*.

*^a^RFD—force developed within the first 50 ms of voluntary muscle contraction on legpress*.

*^b^Average of minimum 12 h/day*.

### Exercise Training and the Clinical State of Patients with Parkinson’s Disease

**Figure 1:**
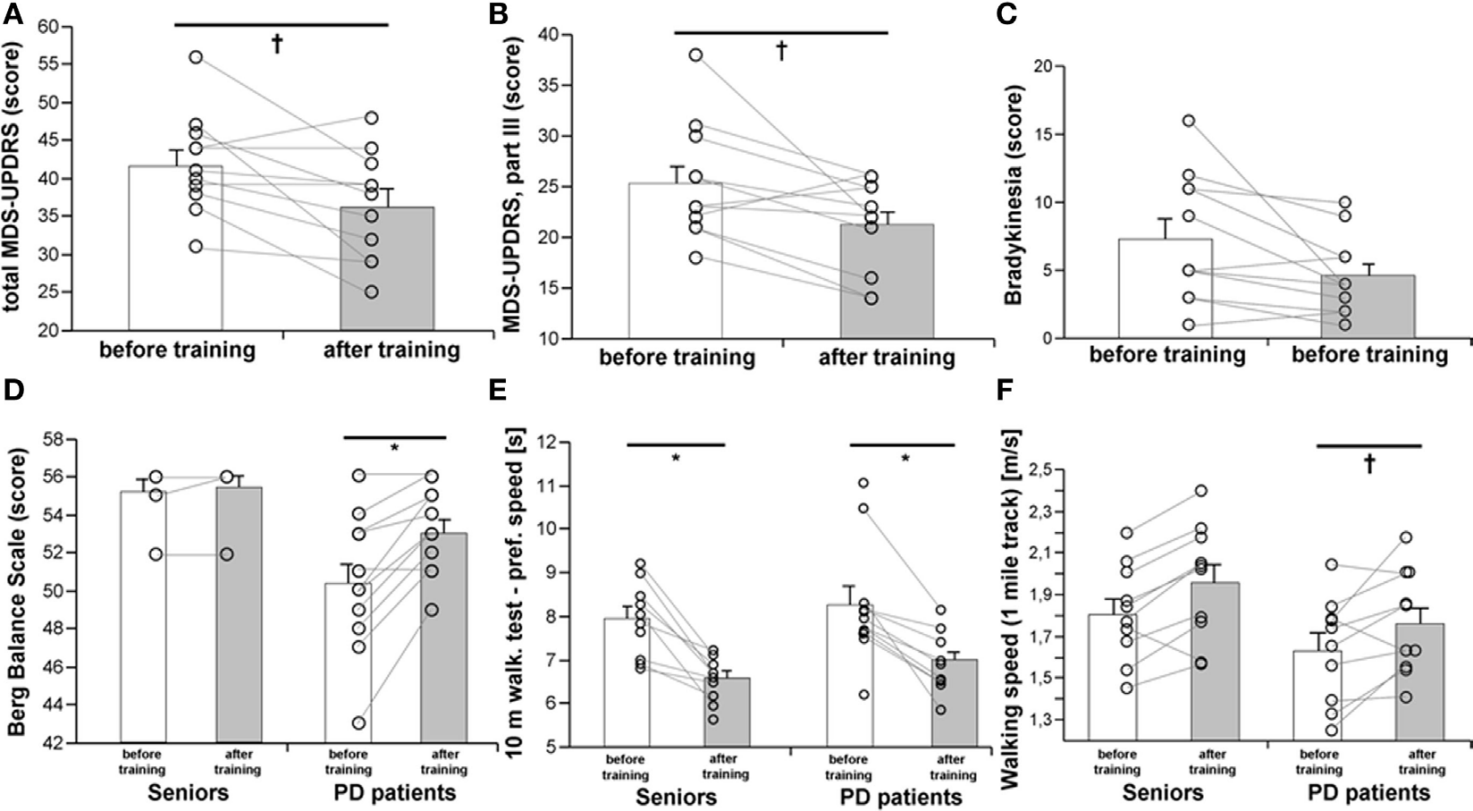
Effects of a 3-month combined endurance/strength training on the clinical state of the PD patients [MDS-UPDRS, **(A,B)**], including bradykinesia **(C)**, balance **(D)**, dynamic motor functions **(E)**, and walking speed on a 1-mile track **(F)**. Data are expressed as average ± SEM, *p < 0.05, ^†^*p* < 0.1.

A 3-month, strength-endurance training program improved the total MDS-UPDRS score (Figure 1A), motor score (Part III), and specifically, the bradykinesia subscore (Figures 1B,C), while no significant improvements in the MDS-UPDRS were found in non-exercising PD patients (Table S1 in Supplementary Material). Exercise benefits on motor functions were also documented by improved static and dynamic balance (BBS), as well as by a better performance in the 10 m preferred speed-walking test, chair-stand-test, and 1-mile Rockport walking test (Figures 1D–F; Table 1). The parameters of balance (BBS) were positively associated with muscle strength (*R* = 0.540, *p* = 0.0036) and VO_2_max (*R* = 0.581, *p* = 0.0008). Exercise training for PD patients had a stronger effect on performance in the maximal speed-walking test (*p* < 0.05). These exercise-induced benefits were present in PD patients despite the fact that the rate of force development was increased only in the control population (Table 1).

### Exercise Training, Metabolic Health, and Physical Fitness

**Figure 2:**
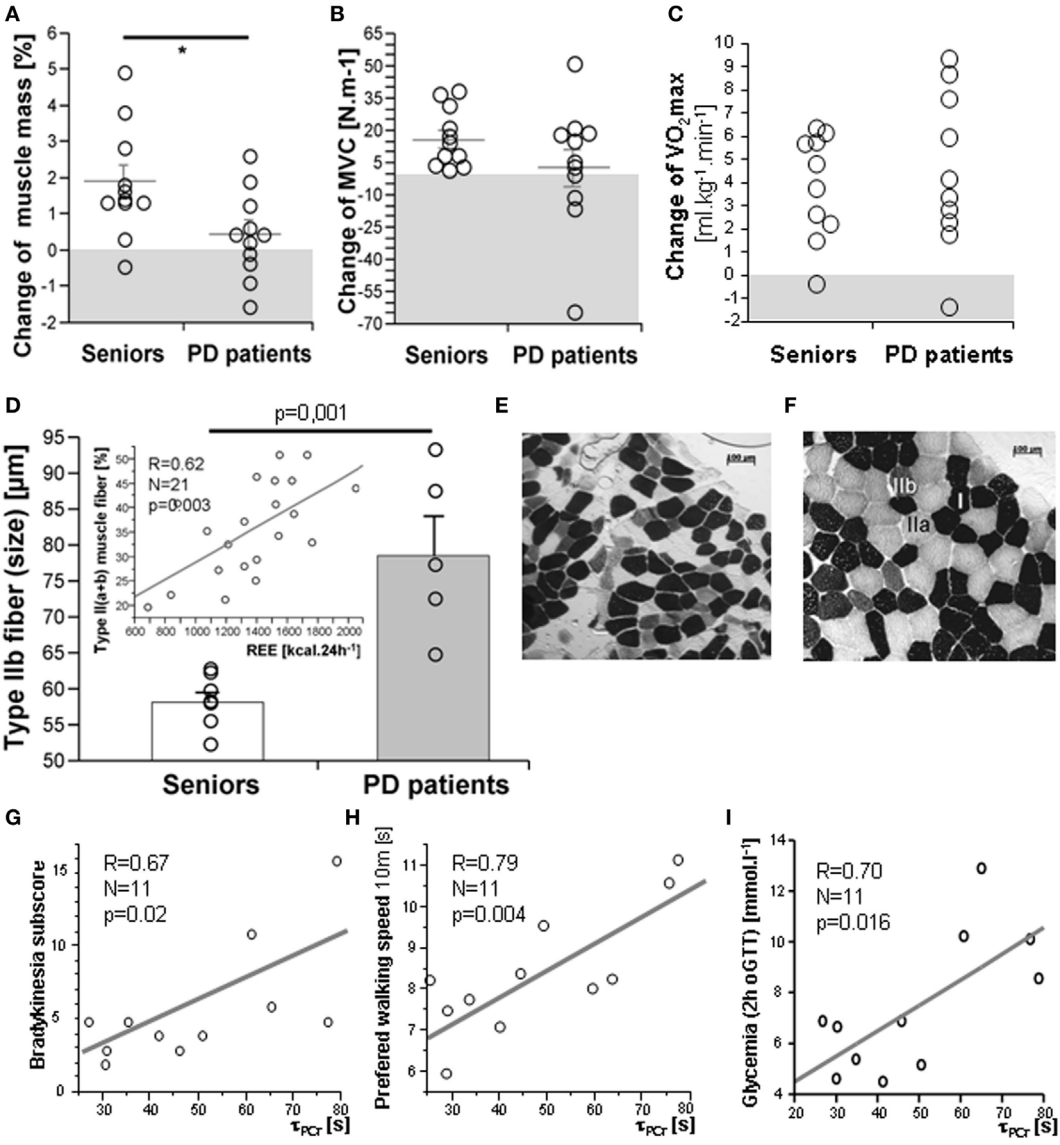
Exercise intervention-induced changes in muscle mass **(A)**, strength **(B)**, and VO_2_max **(C)**. The muscle of PD patients exhibited type IIb fiber hypertrophy **(D,E,F)** and a proportion of type II fibers was associated with resting energy expenditure (REE), [**(D)**-insert], cross-sectional microscopic image of skeletal muscle *(m. vastus lateralis*) from a healthy senior **(E)** and a PD patient **(F)**; Time constant for muscle postexercise phosphocreatine (PCr) recovery (τ_PCr_) was positively associated with the bradykinesia disability score **(G)**, time in the walking test **(H)**, and with 2-h glycemia **(I)**. Data are expressed as average ± SEM, *p < 0.05, ^†^*p* < 0.1; MVC, maximal voluntary contraction force. Type I, IIa, and IIb muscle fibers are identified by different ATPase staining intensity.

**Figure 3:**
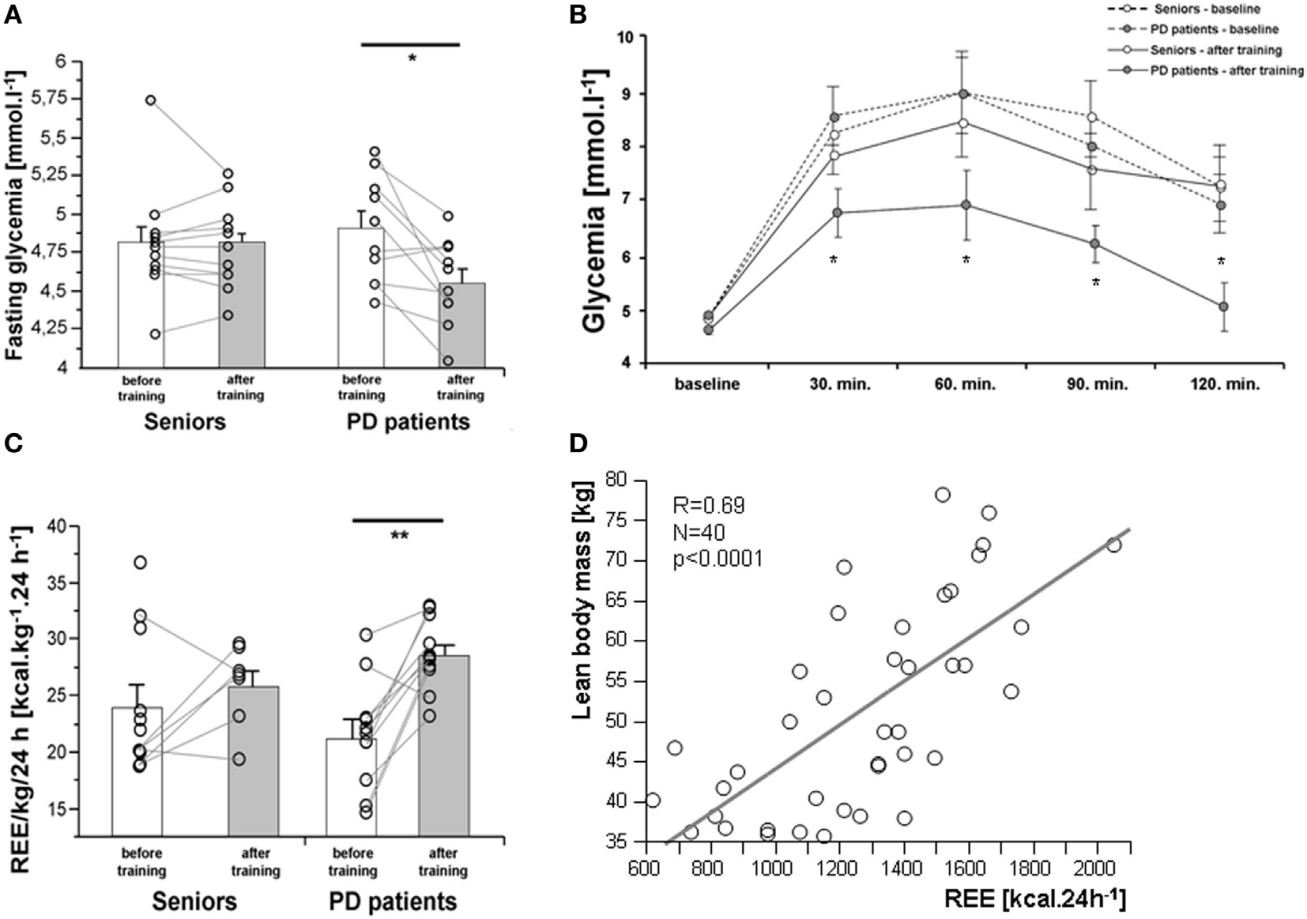
Effects of a 3-month combined endurance/strength training on fasting glycemia **(A)**, glycemic curve [oral glucose tolerance test, **(B)**], and REE **(C)**. REE was associated with muscle mass in the entire study population **(D)**. Data are expressed as average ± SEM, *p < 0.05, ^†^*p* < 0.1; REE, resting energy expenditure; LBM, lean body mass; PD, Parkinson’s disease.

As expected, 3-month exercise training did not induce significant changes in body weight or total body fat mass. However, a trend toward a greater decline in body fat was found in a healthy control population (Table 1). More importantly, the training-induced increase in muscle mass and strength was higher in the control population than in PD patients, who exhibited lower muscle strength compared to controls, both before and after training (Table 1; Figures 2A,B). Exercise significantly increased whole-body REE (Figure 3C) and had a small but consistent lowering effect on RQ, indicating higher exercise-induced metabolic substrate preference for lipids in PD patients than in controls (Table 1). Intervention-induced changes were absent in non-exercising PD patients (*p* = 0.54). Moreover, REE was positively associated with muscle mass (Figure 3D). Compared to controls, PD patients had similar fasting glycemia, but displayed reduced insulin sensitivity (HOMA-IR) in the baseline pre-exercise state (Table 1). Exercise intervention had a greater effect on glucose metabolism in PD patients compared to controls (Table 1), as documented by improved insulin sensitivity, fasting, 2-h glycemia, and area under the glycemic curve (Table 1; Figures 3A,B). Moreover, insulin resistance (HOMA-IR) tended to be negatively associated with maximal aerobic capacity (VO_2_max: *R* = −0.29; *p* = 0.06), as well as with muscle maximal oxidative capacity measured by ^31^P-MRS (Q_max_: *R* = −0.35; *p* = 0.09). Moreover, time needed for muscle postexercise PCr recovery (τ_PCr_) was positively associated with the 2-h glycemia (Figure 2I). Exercise training decreased fasting serum lactate in both PD and healthy control populations (Table 1), and, in PD patients, it reduced serum lactate response to an acute bout of bicycling exercise (Table 1). PD patients tended to be less physically active, with lower levels of aerobic fitness and muscle strength (Table 1). Exercise intervention improved aerobic fitness (VO_2_max) consistently in all individuals (controls/PD patients 13.0/14.2%) (Figure 2C). We observed any intervention-induced improvements in anthropometric and metabolic parameters in the control group of non-exercising PD patients (BMI, *p* = 0.81; body fat, *p* = 0.79; subcutaneous, *p* = 0.77 and visceral adiposity *p* = 0.81; muscle mass, *p* = 0.82; fasting glycemia, *p* = 0.74; 2-h glycemia, *p* = 0.74; fasting insulin, *p* = 0.37; HOMA-IR, *p* = 0.83; hsCRP, *p* = 0.38; total cholesterol, *p* = 0.77; HDL-cholesterol, *p* = 0.8535; TAG, *p* = 0.75).

### Effects of Exercise on Muscle Morphologic, Metabolic, and Molecular Characteristics

**Figure 4:**
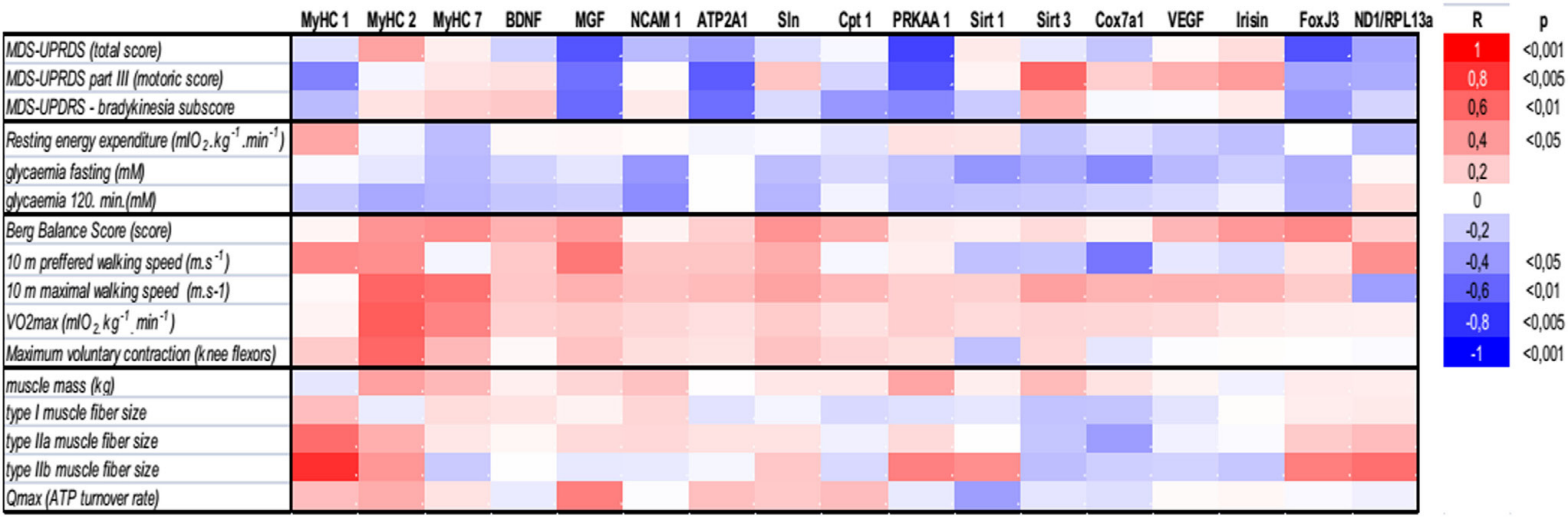
Associations of (i) clinical PD disability score (MDS-UPDRS), (ii) whole-body and muscle metabolism (resting energy expenditure, FPG, 2HG, Qmax), (iii) functional, and (vi) morphological characteristics with the expression of genes related to muscle functional phenotypes, energy metabolism, and mitochondrial biogenesis. ATP2A1, sarcoplasmic/endoplasmic reticulum calcium ATPase 1 isoform (Serca1); BDNF, brain-derived neurotrophic factor; Cpt 1, carnitine palmitoyltransferase 1; Cox7a1, cytochrome *c* oxidase polypeptide 7a1 isoform; FNDC5, fibronectin type III domain containing 5- precursor of Irisin; FoxJ3, forkhead box J3; MGF, mechano-growth factor—splice variant of the Insulin-Like Growth Factor-1 (IGF-1 Ec); MDS-UPDRS, Movement Disorder Society–Unified Parkinson’s Disease Rating Scale, MyHC, myosin heavy chain isoform; NCAM 1, neural cell adhesion molecule 1 isoform; ND1, NADH dehydrogenase subunit 1 was measured to determine the amount of mitochondrial DNA relative to the expression of genomic DNA for Rpl13a; PRKAA1, AMP-activated protein kinase alpha catalytic subunit 1 (AMPKα1); Qmax, dynamic muscle ATP flux; SLN, sarcolipin; Sirt, sirtuin; VEGF, vascular endothelial growth factor; VO_2_max, maximal aerobic capacity.

There were no differences in body composition across the groups, but a small, consistent training-induced increase of muscle mass was found in all control individuals (Table 1). Dynamic ^31^P-MRS showed that τ_PCr_ (the time constant of postexercise PCr recovery) was regulated by training in the muscle of healthy controls, but not in PD patients (Table 1). Moreover, positive associations were found between τ_PCr_ and (i) the bradykinesia subscore (Figure 2G), (ii) the performance in the 10 m preferred speed-walking test (*R* = 0.79; *p* = 0.004), (iii) the 2-h glycemia (Figure 2I), and (iv) the negative association with circulating HDL-cholesterol (*R* = −0.66; *p* = 0.014). Muscle maximal oxidative capacity (Q_max_) was not modified by exercise intervention, but it was associated with bradykinesia (*R* = −0.62; *p* = 0.024) and higher HDL-cholesterol (*R* = 0.51; *p* = 0.05). Compared to controls, the muscle of PD patients contained type IIa and IIb fibers with a larger diameter and their relative content was positively associated with REE (Figures 2D–F). As expected, the glycolytic (type IIb) muscle fiber cross-sectional area was positively associated with MyHC1 mRNA (Figure 4). Regular exercise increased the mRNA of AMPKα1 (PRKAA1) in the skeletal muscle of both controls and PD patients (Table 1), while muscle mitochondrial DNA content and MyHC2/7 ratio (indicative of type II fiber prevalence) were upregulated only in the control population (Table 1). Exercise-related improvements in the PD clinical state (MDS-UPDRS) were associated with increased muscle expression of PRKAA1 and mechano-growth factor (MGF) (Figure 4). Moreover, improvements in fasting glycemia were positively associated with muscle expression of Sirt1 and Cox7a1 (Figure 4). Muscle functional parameters were positively associated with the expression of MyHC2, MyHC7, and MGF (Figure 4).

### Determinants of Exercise-Induced Effects on Fitness and Clinical State in PD

In PD patients, VO_2_max was associated with better performance in the chair-stand test (*R* = 0.54, *p* = 0.012), the maximum walking speed test (*R* = 0.83, *p* = 0.0001), as well as with muscle strength (*R* = 0.71, *p* = 0.0003).

A reduced disability score (MDS-UPDRS) was associated with improvements in muscle strength (*R* = −0.44, *p* = 0.043), motor functions (*R* = −0.39, *p* = 0.078), and glucose tolerance (*R* = −0.547, *p* = 0.0216). Moreover, exercise-induced improvements in bradykinesia were associated with faster postexercise PCr recovery (τ_PCr_) (Figure 2G) and with higher exercise-evoked maximal muscle oxidative capacity (Q_max_, *R* = −0.62, *p* = 0.024). Both the muscle strength (legpress) and motor functions (part-III MDS-UPDRS) of PD patients tended to be associated with a larger diameter of glycolytic-fast type IIa myofibers (strength: *R* = 0.39, *p* = 0.05; part-III MDS-UPDRS: *R* = 0.29, *p* = 0.09). Multiple regression analysis revealed that REE, ambulatory activity, and muscle strength explained 72.5% of the variability in bradykinesia, the most relevant clinical parameter improved by exercise in our PD patients.

## Discussion

Many clinical studies point to a link between Parkinson’s disease (PD), the neurodegenerative disease with salient motor system manifestations, and metabolic disease, demonstrating that >50% of patients with PD are either glucose-intolerant or have type 2 diabetes (T2D) (9, 11). A prospective study with >36,000 T2D patients and >108,000 controls, with a 7.3-year follow-up, showed that the presence of T2D increases the risk of PD by 36% (27). It has been shown that PD patients with T2D have more severe neurological symptoms (28). Treatment of early-stage PD is generally symptomatic, although therapy that offers neuroprotection is likely to be beneficial. Neuroprotective strategies under study include the enhancement of mitochondrial function, insulin sensitivity, anti-inflammatory, and anti-AGE mechanisms (29). Intensive supervised endurance/strength exercise training was used as a physiological therapeutic modality, with the potential to stimulate many of the above mentioned neuroprotective mechanisms, and to ultimately improve the clinical state of patients with early and mid-stage PD. This strategy provides a unique opportunity to improve our understanding of the role of exercise in PD, especially when parallel changes in the whole-body metabolism and muscle metabolic, functional, and molecular state are being examined.

### Endurance/Strength Training Improved the Clinical State of Parkinson’s Disease Patients

This was exemplified by a >16% decrease in the MDS-UPDRS disability score, mostly due to improvements in motor functions, specifically bradykinesia, and corroborates the results of several previous reports (30, 31). Bradykinesia contributes to impaired balance and increases the risk of falls. Training-induced improvement in the Berg balance test in PD patients was similar to a previously reported improvement after a 6-month complex exercise program (32). Furthermore, PD patients displayed better performance in the 10-m preferred-pace walking test, and in walking speed on a 1-mile track (Rockport test). Others have shown that 5 weeks/10 sessions of robotic treadmill training had comparable effects in a similar patient population (30).

### Effects of Training on Metabolic Health and Physical Fitness

Although 3 months of combined intervention might not be sufficiently long to induce significant shifts in body composition, differences in exercise-induced effects on muscle mass between controls (increase) and PD patients (no change) were evident. This was substantiated by a small but uniform increase of muscle strength in elderly controls, which was not observed in PD patients, who, in fact, were inferior to their healthy counterparts in MVC and rate of force development both before and after 3 months of intervention. Ni et al. showed that 3-month, low-load/high-velocity resistance training improved muscle strength in PD patients (31). Combined aerobic/resistance training increased REE, which paralleled a change in muscle mass. Combined exercise caused a small but consistent increase of VO_2_max in all tested individuals, assessed indirectly by the Rockport test, which enabled us to determine the functional capacity of PD patients to walk. This, however, introduced variability related to differences in balance, gait efficiency, muscle strength, and pain tolerance, which could have prevented us from seeing a more robust exercise-response in VO_2_max. O’Leary et al. reported a 14% increase in VO_2_max, assessed by treadmill ergometry in obese elderly individuals, after a 3-month supervised aerobic training (33). An exercise-induced increase in cardiovascular fitness in PD patients was described (34, 35). While Bergen et al. measured VO_2_max directly, as oxygen consumption at a peak exercise load (bicycle ergometry), we and others examined functional performance using the Rockport walking test (34–36).

Exercise intervention reduced fasting and 2-h glycemia and the area under the post-load glycemic curve in PD patients. Moreover, the insulin-sensitizing effect of exercise was more pronounced in PD patients, who were more insulin-resistant prior to exercise intervention. Exercise-induced improvement in insulin resistance tended to correlate with VO_2_max, as well as with muscle maximal oxidative capacity (Q_max_, ^31^P-MRS). Associations between exercise-induced changes in glucose metabolism and VO_2_max were previously reported in a small population of 16 sedentary seniors (33), but information on exercise-related changes in muscle energy metabolism obtained by ^31^P-MRS in PD patients is unprecedented (26, 37). Exercise intervention led to consistent, progressive lowering of fasting serum lactate in both controls and a PD population, which was paralleled by increased physical fitness. It is plausible to think that trained muscles remove lactate from the systemic circulation with higher efficiency (38).

### Effects of Exercise on Muscle Metabolism, Morphology, and Molecular Characteristics

Postexercise PCr recovery (τ_PCr_) was regulated by exercise intervention in the muscle of healthy controls, but not in PD patients. Moreover, exercise-induced changes in τ_PCr_, as well as in Q_max_ (muscle maximal oxidative capacity), were positively associated with improvements in bradykinesia and negatively associated with circulating HDL-cholesterol. τ_PCr_ was also positively related to walking performance and 2-h glycemia, suggesting that the ability to replenish energy during repeated contractions could also be an important determinant of muscle motoric and metabolic state elicited by regular exercise in PD patients. Training increased the mRNA expression of AMPKα1 (PRKAA1) in the skeletal muscle of controls and PD patients. However, muscle mitochondrial DNA content, type IIa fiber size, MVC force, and muscle mass only increased in the control population, indicating differences in the magnitude of exercise-induced adaptive changes in the muscle of PD patients. Moreover, exercise-related improvements in the PD disability score (MDS-UPDRS) were associated with the increased muscle expression of AMPKα1 and MGF, indicating the importance of both the metabolic and the mitogenic response. There is a line of evidence that dysregulation of mitochondrial function and energy metabolism are the key determinants of PD pathology, as the maintenance of mitochondrial homeostasis is crucial for neuronal development (39). The induction of PGC-1α, a regulator of mitochondrial biogenesis, *via* activation of PPARs (rosiglitazone, bezafibrate), or the modulation of energy metabolism by activating AMPK (AICAR, metformin, resveratrol) or Sirt1 (SRT1720, isoflavone-derived compounds), could be highlighted as a potential strategy with which to modulate mitochondrial biogenesis, with effects similar to those of exercise (39). Various exercise benefits had, in our hands, distinct gene predictors. Reduced fasting glycemia was positively associated with the expression of Sirt1 and Cox7a1 and muscle functional parameters were positively associated with the expression of MyHC2, MyHC7, and MGF, indicating the importance of mechanical sensing and fiber type shift in response to regular muscle use. Our results are in agreement with an observation that a single bout of sprint-induced mitochondrial activity was associated with the increased expression of Sirt1, the upstream-deacetylase of PGC-1α, and the induction of Thr(172) AMPKα phosphorylation (40). We observed that type IIb and IIa fibers with an enlarged cross-sectional area represented the major morphological characteristic of muscle in our PD patients, while the size of type I fibers did not differ between the groups. This is in agreement with a higher expression of *MyHC1* and *MyHC2* genes (markers of type IIa and IIb fibers) and a lack of change in *MyHC7* (type I fiber marker). Regular exercise increased the expression of *MyHC1* in controls, but not in a PD population, with *MyHC7* expression unregulated. The shifts in muscle morphology/phenotype that occur in PD may come about as a consequence of the modified pattern of motor unit activation and rigidity. Previous histochemical studies demonstrated both atrophic and hypertrophic fibers and fiber type grouping in the limb muscles of PD patients (41, 42). Histological changes in muscle fiber composition appear to be muscle-specific and related to PD subtypes (41). It is plausible to think that shifts in muscle fiber composition in PD are related to duration, severity, and the specific clinical character of the disease. Our results showed that exercise lowered the type 1/type 2 ratio in both controls and the PD group, likely resulting from either type 2 fiber hypertrophy or hyperplasia, induced by endurance/strength training.

### Determinants of Exercise-Induced Effects on Physical Fitness and the Clinical State of PD

Preliminary evidence indicates that regular physical activity has the capacity to target both motor and metabolic dysfunction in PD patients. However, the mechanisms and determinants of the interplay between neurodegeneration and metabolism are unexplored. A sedentary lifestyle and chronic energy overload are common denominators of neurodegeneration and metabolic impairment (43), promoting chronic inflammation and insulin resistance, both at the whole-body level and in the brain (13, 44). In our study, we observed increased peripheral IR in PD patients, pointing at systemic metabolic dysfunction in PD compared to age/BMI-matched controls, which was alleviated by exercise. It can be assumed that systemic insulin resistance is mirrored by brain insulin resistance, which has been linked to compromised cholesterol synthesis and mitochondrial function, decreased brain plasticity, and apoptosis (45).

Our preliminary data, indicating a positive association between serum and CSF glucose (2PD/6controls), support the notion that exercise modulates both peripheral and central glucose metabolism, which has been thought to counteract the neurodegenerative process (46).

Moreover, muscle maximal oxidative capacity and/or the time needed for postexercise PCr recovery were associated with muscle functional parameters, REE, and whole-body glucose metabolism. Recent advances in MR spectroscopy have enabled us to study both muscle and brain function and metabolism *in vivo*, providing detailed compartmentalized information about metabolic interactions between neurons (47) and about the levels of important neurotransmitters (GABA, glutamate) (48).

We and others have shown that reduced muscle mitochondrial content/function is linked to insulin resistance and T2D (49, 50). However, regular exercise is a well-recognized stimulus of mitochondrial biogenesis, not only at the level of skeletal muscle (51), but also at the systemic level (52). In this study, we have shown that exercise failed to upregulate muscle mitochondrial DNA content in PD patients compared to controls. However, an increase in the expression of *AMPK*α*1* indicates that signaling pathways involved in mitochondrial biogenesis, as well as glucose uptake, contributed to the adaptive response to exercise (53). Further research is necessary to evaluate the impact of exercise on muscle metabolism and mitochondrial content/function in PD.

In conclusion, complex intensive, although relatively short endurance-strength exercise intervention, improved the clinical state of early/mid-stage PD patients. This was associated with improvements in muscle and whole-body metabolism, suggesting that life-long maintenance of muscle metabolic and functional capacity by regular exercise is essential to counteract the pathophysiology of PD and should become a part of the management for Parkinson’s disease.

## Ethics Statement

This study was carried out in accordance with the Declaration of Helsinky. All subjects provided written informed consent prior entering the study. The protocol was approved by the “Ethics Committee of Faculty of Medicine, Comenius University and Univesrity Hospital Bratislava.”

## Author Contributions

Project conception and organization (BU, JU, AT); clinical examinations (PK, LS, MS, IS, SS, PT, PV, JU, BU); exercise intervention design and execution (VT, LS, MV, JC, MS); MR measurements (RK, LV, MK); histological examinations (ZJ, JU); muscle molecular analyses (PK, SV); and manuscript writing (PK, JU, BU).

## Conflict of Interest Statement

The authors declare that the research was conducted in the absence of any commercial or financial relationships that could be construed as a potential conflict of interest.
