# Supplementary figures and images for: Aerobic-Strength Exercise Improves Metabolism and Clinical State in Parkinson’s Disease Patients
Source: Front Neurol. 2017 Dec 22;8:698. doi: 10.3389/fneur.2017.00698 (PMC5743754; doi:10.3389/fneur.2017.00698)

# Supplementary figure 1

## Clinical study flow chart

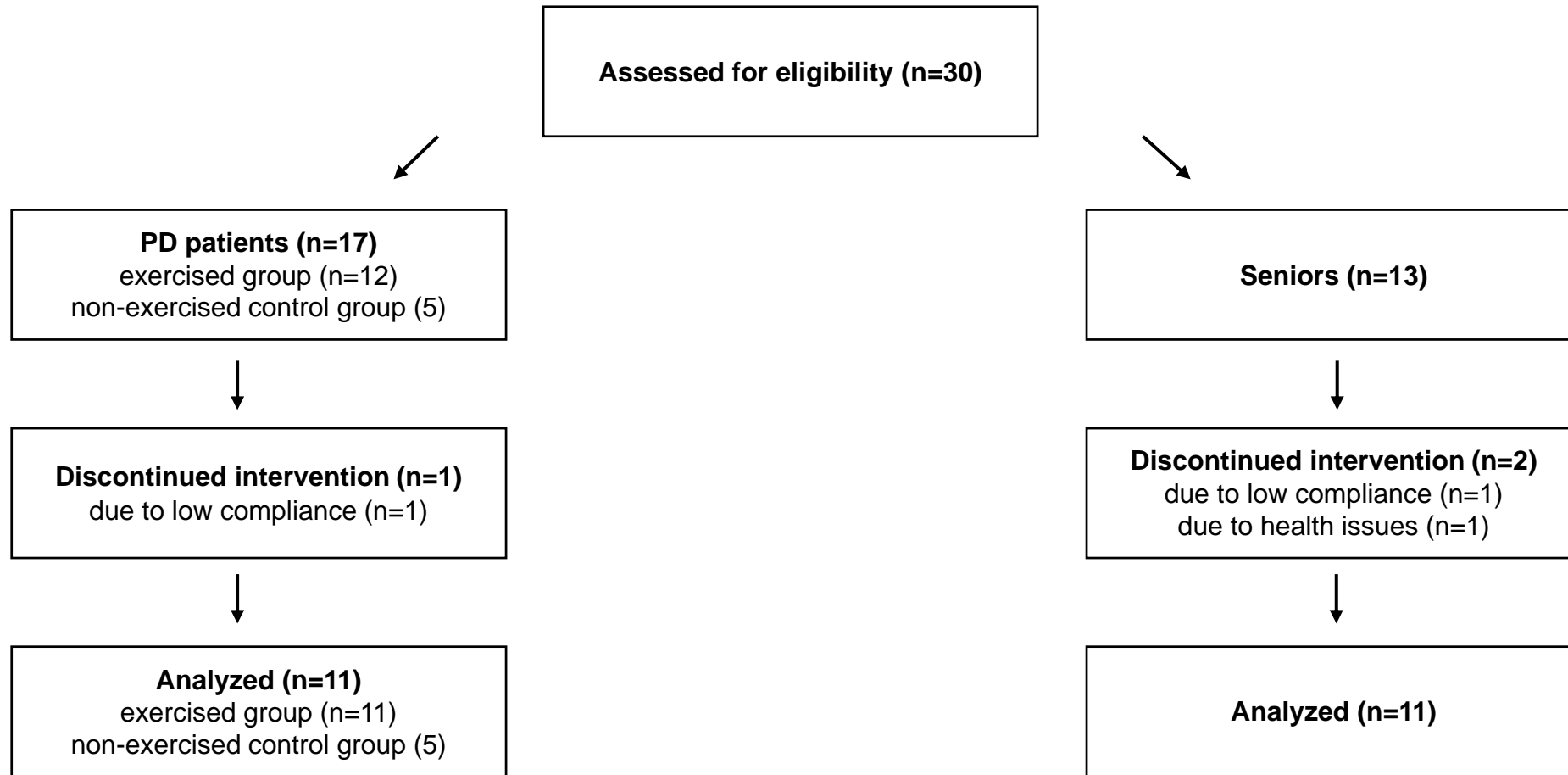

Supplement: Supplementary file 1 [file Image_1.PDF]
