# Supplementary material for: Aerobic-Strength Exercise Improves Metabolism and Clinical State in Parkinson’s Disease Patients
Source: Front Neurol. 2017 Dec 22;8:698. doi: 10.3389/fneur.2017.00698 (PMC5743754; doi:10.3389/fneur.2017.00698)
Supplement: Supplementary file 2 [file Table_1.doc]

Supplementary table 1

**Specific exercise-induced changes in MDS-UPDRS**

* - used to calculate bradykinesia subscore, expressed in % or as number of patients with the specific symptom within the population. Abbreviations: DDS – dopamine dysregulation syndrome; LLE – left lower extremity; LUE – left upper extremity; RLE – right lower extremity; RUE – right upper extremity

|  | **description** | before training | after training |
| --- | --- | --- | --- |
| **Part I** |  | 44,8% | 43,4% |
| 1.1 | cognitive impairment | 4/11 | 4/11 |
| 1.2 | hallucination and psychosis | 0/11 | 1/11 |
| 1.3 | depressed mood | 5/11 | 4/11 |
| 1.4 | anxious mood | 5/11 | 8/11 |
| 1.5 | apathy | **8/11** | **3/11** |
| 1.6 | features of DDS | 1/11 | 0/11 |
| 1.7 | sleep problems | 8/11 | 5/11 |
| 1.8 | daytime sleepiness | 6/11 | 4/11 |
| 1.9 | pain and other sensations | 8/11 | 7/11 |
| 1.10 | urinary problems | 5/11 | 7/11 |
| 1.11 | constipation problems | 4/11 | 6/11 |
| 1.12 | light headedness | 1/11 | 3/11 |
| 1.13 | fatigue | 7/11 | 9/11 |
| **Part II** |  | 39,9% | 39,9% |
| 2.1 | speech | 6/11 | 6/11 |
| 2.2 | saliva and drooling | 3/11 | 4/11 |
| 2.3 | chawing and swallowing | 1/11 | 3/11 |
| 2.4 | eating tasks | 4/11 | 3/11 |
| 2.5 | dressing | 6/11 | 6/11 |
| 2.6 | hygiene | 3/11 | 3/11 |
| 2.7 | handwriting | 8/11 | 6/11 |
| 2.8 | doing hobbies and other activities | 3/11 | 2/11 |
| 2.9 | turning in bed | 6/11 | 5/11 |
| 2.10 | tremor | 5/11 | 5/11 |
| 2.11 | getting out of bed | 3/11 | 5/11 |
| 2.12 | walking and balance | 6/11 | 7/11 |
| 2.13 | freezing | 3/11 | 2/11 |
| **Part III** |  | 54,0% | 49,3% |
| 3.1 | speech | 8/11 | 9/11 |
| 3.2 | facial expression | 10/11 | 10/11 |
| 3.3 (a-e) | rigidity – neck, RUE, LUE, RLE, LLE | 96% | 100% |
| 3.4 (a-b) ***** | finger tapping – right hand, left hand | **77%** | **45%** |
| 3.5 (a-b) ***** | hand movements – right hand, left hand | **50%** | **36%** |
| 3.6 (a-b) ***** | pronation-supination movements – right, left hand | **45%** | **36%** |
| 3.7 (a-b) ***** | toe tapping – right foot, left foot | 72% | 77% |
| 3.8 (a-b) | leg agility – right leg, left leg | **82%** | **68%** |
| 3.9 | arising from chair | 0/11 | 0/11 |
| 3.10 | gait | 11/11 | 10/11 |
| 3.11 | freezing of gait | 2/11 | 2/11 |
| 3.12 | postural stability | 1/11 | 1/11 |
| 3.13 | posture | 10/11 | 11/11 |
| 3.14 | global spontaneity of movement | 9/11 | 10/11 |
| 3.15 (a-b) | postural tremor – right hand, left hand | **32%** | **18%** |
| 3.16 (a-b) | kinetic tremor – right hand, left hand | 14% | 18% |
| 3.17 (a-e) | rest tremor – RUE, LUE, RLE, LLE, lip/jaw | **11%** | **5%** |
| 3.18 | constancy of rest | 4/11 | 2/11 |
| **Part IV** |  | 21,2% | 16,7% |
| 4.1 | time spent with dyskinesias | 2/11 | 3/11 |
| 4.2 | functional impact of dyskinesias | 2/11 | 2/11 |
| 4.3 | time spent in the OFF state | 3/11 | 2/11 |
| 4.4 | functional impact of fluctuations | 4/11 | 2/11 |
| 4.5 | complexity of motor fluctuations | 3/11 | 2/11 |
| 4.6 | painful OFF-state dystonia | 0/11 | 0/11 |
