# Supplementary material for: Aerobic-Strength Exercise Improves Metabolism and Clinical State in Parkinson’s Disease Patients
Source: Front Neurol. 2017 Dec 22;8:698. doi: 10.3389/fneur.2017.00698 (PMC5743754; doi:10.3389/fneur.2017.00698)
Supplement: Supplementary file 3 [file Table_2.doc]

Supplementary table 2

**Primer sequence of genes related to energy metabolism, fibertype and mitochondrial content**

ND1, NADH dehydrogenase subunit 1; Rpl13a, Ribosomal protein L13a; MyHC, myosin heavy chain isoform; BDNF, brain-derived neurotrophic factor; MGF, mechano growth factor - splice variant of the Insulin-Like Growth Factor-1 (IGF-1 Ec); NCAM 1, neural cell adhesion molecule 1 isoform; ATP2A1, sarcoplasmic/endoplasmic reticulum calcium ATPase 1 isoform (Serca1); Sln, sarcolipin; Cpt 1, carnitine palmitoyltransferase 1 isoform; PRKAA1, AMP-activated protein kinase alpha catalytic subunit 1 (AMPKα1); Sirt, sirtuin isoform; Cox7a1, cytochrome c oxidase polypeptide 7a1 isoform; VEGF, vascular endothelial growth factor; FNDC5, fibronectin type III domain containing 5- precursor of Irisin.

| Gene | forward (fwd) primer sequence | reverse (rev) primer sequence |
| --- | --- | --- |
| MT-ND1 | CCCTAAAACCCGCCACATCT | GAGCGATGGTGAGAGCTAAGG |
| Rpl13a | GGACCGTGCGAGGTATGCT | ATGCCGTCAAACACCTTGAGA |
| 18S | CGGACCAGAGCGAAAGCAT | CCTCCGACTTTCGTTCTTGATT |
| MyHC1 | TAAGACCGAGGCAAAAAGGA | TGCATCAGCCAAGCTGTC |
| MyHC2 | TGTCTCACTCCCAGGCTACA | CCAAAAACAGCCAATTCTGAG |
| MyHC7 | CTTCGTGCCTGATGACAAACA | CACGGTCACTGTCTTGCCATA |
| BDNF | GTGCCGAACTACCCAGTCGTA | TCGCCAGCCAATTCTCTTTT |
| MGF | CGAAGTCTCAGAGAAGGAAAGG | ACAGGTAACTCGTGCAGAGC |
| NCAM 1 | AGACCCCATTCCCTCCAT | ATGTGCCCATCCAGAGTCTT |
| ATP2A1 | GGATGGGCCTCATGTCAACTA | CTATGCCCTCAAAGTGGGTGTT |
| Sln | GCCATAGCCAGGGTGTGTCT | TCTGAGGGCACACCAAGGA |
| Cpt 1 | GGAAACGGCCAACTGCAT | ACAGGCCGGTGCCAAAC |
| PRKAA1 | TCTCAGGAGGAGAGCTATTTGATT | GAACAGACGCCGACTTTCTTT |
| Sirt 1 | CCTCACATGCAAGCTCTAGTGACT | CAATCATAAGATGTTGCTGAACAAAA |
| Cox7a1 | TGACATCCCGTTGTACCTGAAG | ACAGTGCCGCCCAGACA |
| VEGF | TCATCACGAAGTGGTGAAGTTCA | TCAGGGTACTCCTGGAAGATGTC |
| Irisin/FNDC5 | TGAGGTTGTCATCGGATTTGC | GCGGGTGGTGGTGTTCAC |
